# Focus Harmonic Scalpel Compared to Conventional Haemostasis in Open Total Thyroidectomy: A Prospective Randomized Trial

**DOI:** 10.1155/2011/357195

**Published:** 2011-09-29

**Authors:** Emanuele Ferri, Enrico Armato, Giacomo Spinato, Roberto Spinato

**Affiliations:** ^1^Otorhinolaryngology Department ULSS 13, General Hospitals of Dolo and Mirano, Via Mariutto 76, 30035 Mirano, Venice, Italy; ^2^ENT Clinic, Head and Neck Department, University of Trieste, Hospital of Cattinara, Strada di Fiume 447, 34149 Trieste, Italy

## Abstract

The aim of this prospective randomized trial was to compare operative factors, postoperative outcomes and surgical complications of open total thyroidectomy when using the Harmonic Scalpel (HS) versus Conventional Haemostasis (CH). *Methods*. 100 consecutive patients underwent open total thyroidectomy were randomized into two groups: group CH (Conventional Haemostasis) and group HS (Harmonic Scalpel). We recorded the following: age, sex, pathology, thyroid volume, haemostatic technique, operative time, drainage volume, thyroid weight, postoperative pain, postoperative complications, and hospital stay. The results were analyzed using the Student's *t *test and *χ* ^2^ test. *Results*. No significant difference was found between the two groups concerning mean thyroid weight and mean hospital stay. The mean operative time was significantly shorter in the HS group. The total drainage fluid volume was lower in HS group. Two (4%) transient recurrent laryngeal nerve palsies were observed in CH group and no one (0%) in the HS group. Postoperative transient hypocalcemia occurred more frequently in the CH group. HS group experienced significantly less postoperative pain at 24 and 48 hours. *Conclusions*. In patients undergoing thyroidectomy, HS is a reliable and safe tool. Comparing with CH techniques, its use reduces operative times, postoperative pain, drainage volume and transient hypocalcemia.

## 1. Introduction

The pioneers of thyroid surgery, Theodor Kocher and Theodor Billroth, developed an acceptable technique of standardized thyroid surgery between 1873 and 1883. By 1920, the principles of safe and efficient thyroid surgery were already established [1]. They consist of three basic phases: identification and ligation of vessels, identification and preservation of laryngeal nerves, and parathyroid glands. Basic surgical instruments are not significantly changed; the main innovations are new methods of coagulation and vascular section.

During the last decade, alternative techniques for improving safety, effectiveness, and even invasiveness of thyroidectomy have been proposed, including video-assisted, and endoscopic surgery, nerve monitoring, and less invasive forms of anesthesia [2]. Bleeding remains one of the major postoperative complications of thyroid surgery, with the potential to cause life-threatening airway obstruction. During thyroidectomy, bleeding can obscure the operative field, making safe dissection of the recurrent laryngeal nerve (RLN), and parathyroid glands difficult. Effective vessel haemostasis can be achieved by using the conventional clamp-and-tie technique. Newer techniques of vessel haemostasis hope to be more rapid while achieving the same effectiveness [3]. Several studies have reported the successful use of bipolar vessel sealing systems [4] or the HS [5] in shortening the length of thyroid surgery and reducing blood loss, while retaining a good safety profile. However, these techniques do incur the cost of generators and hand pieces, which may be difficult to justify in some departments [6]. It has been claimed that the use of the HS decreases operative time, complications and bleeding in abdominal surgery [7], thoracic surgery [8], parotid surgery [9], and thyroid surgery [10].

The present prospective randomized trial study was designed to evaluate the efficacy and safety of HS use compared with conventional haemostasis (CH) in open thyroid surgery. The primary objectives of this study were the reduction of operative time, postoperative pain and overall drainage volume in thyroid surgery with the use of the HS. The secondary objective was the comparison between groups of hospital stay and surgical complications in thyroidectomy, such as hypocalcemia and RLN palsy.

## 2. Patients, Materials, and Methods

Between January 2010 and May 2011, at the Otorhinolaryngology Department of General Hospital of Mirano (Venice, Italy), 100 consecutive patients with benign or malignant thyroid disease underwent open total thyroidectomy performed by the same team of surgeons with remarkable experience in the thyroid surgery. Patients were randomly assigned to either the HS group (50 patients in which the operation was performed entirely using the HS and no other haemostatic tool) or the CH group (50 patients in which the operation was performed using CH tools such as the classic technique of tying and knots, resorbable ligature, bipolar diathermy) (Table 1).

**Figure 1:**
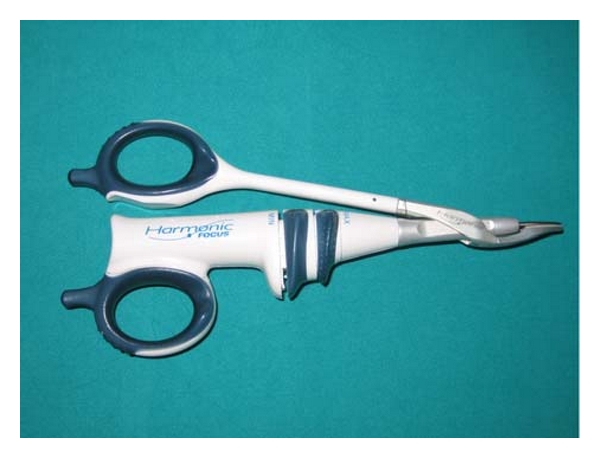
Focus Ultracision Harmonic Scalpel (Ethicon Endo-Surgery, Inc, Cincinnati, Ohio, USA).

**Figure 2:**
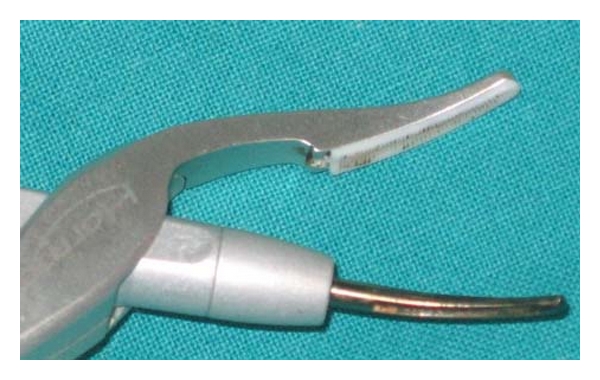
The curved blade and the clamp arm with Teflon pad of the Focus Ultracision Harmonic Scalpel used in otolaryngologic surgery.

We used the Focus Ultracision Harmonic Scalpel (Ethicon Endo-Surgery, Inc, Cincinnati, Ohio, USA) (Figure 1). The harmonic scalpel setup consists of a generator, a hand piece and a blade. The hand piece contains an ultrasonic transducer that consists of a stack of piezoelectric crystals sandwiched between two metal cylinders under pressure. The transducer is attached to the blade through a mount. The 110-volt generator is a high-frequency switching power supply controlled by a microprocessor that pulses the transducer in the hand piece with AC current. This current allows the transducer to vibrate at its natural harmonic frequency of 55.5 kHz. The blade used most frequently in otolaryngological procedures looks like a curved paddle with a sharp inner beveled side for cutting and a blunt outer radius for coaptive coagulating (Figure 2). The generator can be adjusted from a level of 1 to 5 to increase cutting speed and decrease coagulation by increasing the blade's lateral excursion [11–14].

All patients were blinded to the surgical technique used and signed an informed consent before enrollment in the trial. The patients were divided according to age, preoperative diagnosis, and thyroid size to generate homogeneous groups. The inclusion criteria were: (1) age >18 years, (2) acceptance to participate in the study (signed informed consent form), and (3) scheduled total thyroidectomy for multinodular goiters or low risk differentiated carcinoma (T1N0M0). The exclusion criteria were: (1) preoperative medication including analgesics, corticosteroids or nonsteroidal antiinflammatory drugs; (2) coagulation disorders; (3) pregnancy; (4) cervicomediastinal goiters; (5) total thyroidectomy with need of lymph node block dissection as in patients with malignant invasive cancer; (6) concomitant parathyroid disorders; (7) previous neck surgery and (8) history of neck irradiation.

**Figure 3:**
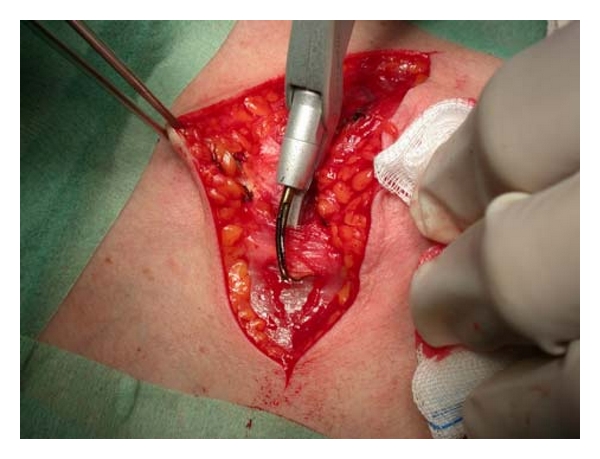
Dissection of the muscle platysma with Focus Ultracision Harmonic Scalpel.

**Figure 4:**
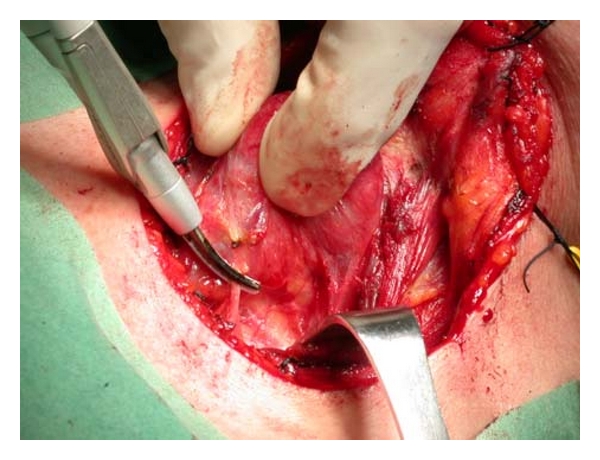
Sealing of inferior thyroid artery with Focus Ultracision Harmonic Scalpel.

**Figure 5:**
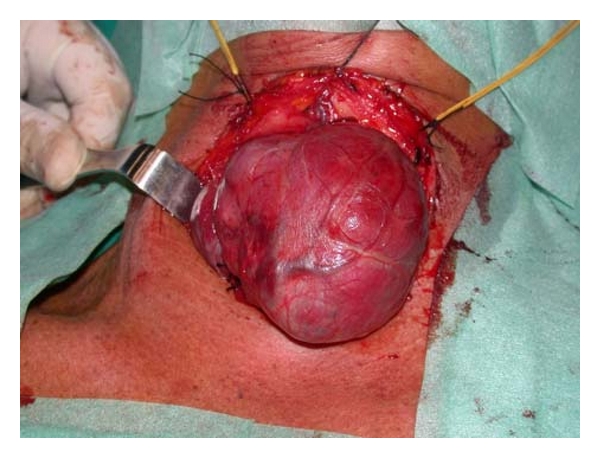
Postoperative thyroid specimen (multinodular goiter).

A total thyroidectomy for benign or malignant low-risk thyroid disease (as papillary carcinoma T1N0M0) was performed under general anesthesia and with endotracheal intubation in all cases. A complete preoperative assessment (serum thyrotropin levels, ultrasonography to evaluate nodule size and gland volume, and fine-needle aspiration cytology) was obtained for all patients; they were positioned and draped in the conventional manner. A 4 to 6 cm incision (depending on the size of the thyroid) was made over the level of the thyroid isthmus. Subplatysmal flaps were developed, and the strap muscles were separated in the midline and laterally reflected. The inferior, middle, and superior thyroid vessels were then divided either with the HS or with conventional technique. The thyroid lobe was then medially rotated, and the vessels in the ligament of Berry, with the RLN under direct vision, were clamped and tied in both groups. The same steps are repeated for removal of the contra lateral lobe. Finally, the wound was irrigated and closed using interrupted 3-0 polyglactin sutures (Vycril, Ethicon) to approximate the strap muscles and the platysmal layer. The skin was closed using metal clips (Figures 3, 4, and 5).

 Outcomes of the study included operative time, fluid content in the suction balloon (drainage volume) during the first 24 hours after surgery, postoperative pain, hospital stay, and incidence of complications (rate of hypocalcemia and RLN injury). Suction drainage was used to evaluate the overall amount of blood loss after the procedure and to assess the actual difference between the groups. The drains were removed 24–48 hours after surgery. Both preoperative and postoperative RLN statuses were determined by indirect laryngoscopy. In all patients, serum calcium levels were obtained during the first postoperative day and then once every 3 weeks. Patients with low calcium levels on the first postoperative day were asked to return the next day to have the level rechecked. Patients were given acetaminophen, 1000 mg every 8 hours, for the first 24 hours after surgery. Pain assessment was analyzed according to patient responses to a visual analogic scale (VAS) and a verbal response scale (VRS). Anesthesiologists completely unaware of the surgical instrumentation used during the procedure collected all data relative to postoperative pain. The VAS consisted of a printed 10 cm horizontal line anchored by the descriptors “no pain” (minimum, on the left end of the scale) and “worst pain imaginable” (maximum, on the right end). All subjects were in good general health, had no known neurological disorders, and were taking no medications. Patients used the VAS to assess their level of pain when they started deglutition and early feeding (generally 24 and 48 hours after the operation). They were also asked to describe the anatomical location of the pain, in particular to differentiate postoperative surgical incision pain from back or neck pain not due to the surgical procedure. To avoid any setting bias, the clinician always moved the scale's indicator to the horizontal midpoint before the instrument was handed to the patient for a response. The VRS offered 5 options: 0 for no; 1, light; 2, endurable; 3, strong; and 4, unendurable pain. The patients graded their pain at 24 and 48 hours after surgery.

Patients were also asked to contact the Department of Otorhinolaryngology at the General Hospital of Mirano (Venice, Italy) after discharge for any postoperative complication such as neck hematoma or seroma and wound infection. The ethical committee of the Surgical Department approved the study protocol. All patients gave informed written consent. The results were analyzed using the Student's *t-*test and *χ* ^2^ test. A value of *P* < .05 was considered statistically significant.

## 3. Results

The demographic characteristics of the patients and the preoperative diagnosis are showed in Table 1. No significant difference was found between the two groups concerning mean thyroid weight (44.8 ± 19.7 grams in HS group; 51.1 ± 15.8 grams in CH group; *P* > .05) and mean hospital stay (2.2 ± 0.9 days in HS group; 3.7 ± 1.3 days in CH group; *P* > .05) (Tables 1 and 2).

The average operative time was significantly shorter in the HS group (44.9 ± 8.3 minutes) compared with the CH group (69.5 ± 10.7 minutes; *P* < .001) (Table 2). The total drainage fluid volume was lower in HS than in CH (37.4 ± 2.4 versus 56.1 ± 4.2, resp., *P* < .001) (Table 2).

Complications rate was observed in both groups. Two (4%) transient RLN palsies were observed in CH group and no one (0%) in the HS group. No patient developed permanent palsy (*P* = *NS*) (Table 2). Postoperative transient hypocalcemia occurred more frequently in the CH group than in the HS group. This difference was statistically significant (21/50, 42% in CH group; 7/50, 14% in HS group) (*P* < .01). Hypocalcemia was defined as a serum calcium level below 8.0 mg/dL (2.00 mmol/L) (reference range, 8.0–10.5 mg/dL [2.00–2.60 mmol/L]). In the CH and HS groups, 19 patients (38%) and 5 patients (10%), respectively, required oral calcium carbonate supplementation postoperatively, because of these patients showed clinical symptoms of hypocalcemia. The lowest serum calcium level was 7.5 mg/dL (1.87 mmol/L) in the CH group versus 7.9 mg/dL (1.96 mmol/L) in the HS group. All patients recovered completely and no definitive hypoparathyroidism was registered (Table 2).

According to the VAS and VRS scores, patients of the HS group experienced significantly less postoperative pain compared with patients of the CH group. The differences in VAS scores between the HS and CH groups were statistically significant at 24 and 48 hours (*P* < .001). The difference in the VRS score between the groups was statistically significant at 24 and 48 hours after surgery (*P* < .001) (Table 2).

## 4. Discussion

Total thyroidectomy is a surgical procedure that requires meticulous dissection, safe anatomical exposure, and effective hemostasis. Total thyroidectomy is the treatment of choice for many thyroid diseases. This operation is performed frequently, with no mortality and low morbidity. Morbidity mainly results from postoperative laryngeal nerve palsy (transitory or definite) and hypocalcemia (clinical or nonclinical, transitory or definite). Incidence of RLN palsies varies from 0% to 23%, whereas transient asymptomatic hypocalcemia after total thyroidectomy may reach 63% [15].

The HS is a new device that has been introduced to surgery during the last decade. It uses high frequency mechanical energy to cut and coagulate tissues at the same time. Ultrasonic coagulation achieved by the HS is similar to that of electrocautery in that the ultimate result remains a denatured protein coagulum that coapts and tamponades blood vessels. However, the mechanism by which the proteins become denatured is completely different. Both electrocautery and lasers form the coagulum by heating tissue to denature the protein. The HS denatures protein by using ultrasonic vibration to transfer mechanical energy sufficient to break tertiary hydrogen bonds [11]. At least two mechanisms exist by which the HS cuts: cavitational fragmentation and mechanical cutting. The blade vibrates at 55.5 kHz over a distance of 80 *μ*m [12]. In a porcine study comparing vessel-sealing systems using various modalities of energy, including the HS, the LigaSure vessel sealing system (Valleylab, Boulder, Colorado), and two types of bipolar forceps, the HS was found to seal arteries 3.8 mm in diameter on average and veins 9.9 mm in diameter on average. This sealing ability was essentially inferior to that of the other systems. However, the HS showed a smaller area of lateral thermal damage compared to the bipolar cautery [13].

The HS was originally developed for its applications in laparoscopic abdominal surgery but has found a successful application into otolaryngology specialty [16]. The primary application for the HS in the otolaryngological literature is its use for tonsillectomy and thyroidectomy. The use of the HS has also been described in excising cancer of the tongue and soft palate [17], submandibular sialadenectomy [18], parotidectomy [9, 19, 20], treating allergic rhinitis by means of inferior turbinate alteration [21], and surgical treatment of rhinophyma [22]. Unlike the variable results described with the use of the HS in tonsillectomy, literature is consistent concerning the usefulness of the harmonic scalpel in thyroid surgery. Operative times are consistently lower, bleeding is insignificant, and the resulting cost containment is evident. In addition to the shorter operating time, Shemen reports the advantage of a smaller incision (4.5 versus 5.5 cm) [23]. Vach et al. report that pathologists can more easily evaluate thyroid specimens obtained with the HS [24]. In their work with video-assisted thyroidectomy, Miccoli et al. confirmed the significant reduction in operative time and a lack of complications in the HS group [25]. Finally, in a prospective, randomized study of 200 patients, Ortega et al. confirmed a 15% to 20% reduction in operative time [26].

During thyroidectomy, the dissection, ligation, and division of the major thyroid vessels are time consuming. We showed it is possible to shorten operative time by using HS. Statistical analysis showed operative time was shorter in operations where a HS was used to seal the small vessels of the thyroid gland. Operative time was further shortened when HS was used for all of the vessels (including main arteries and veins). Bleeding in thyroid surgery can occur from the main arteries and veins of the gland (a.v. thyroidea inf, sup), small tributaries, or the gland itself (due to inappropriate dissection resulting in ligation and inadvertent traction). The Ultracision HS has been approved by the United States Food and Drug Administration for the ligation of vessels up to 3 mm in diameter. Thermal damage is limited to 0–2 mm beyond the tissue grasped within the forceps of the device [27–29]. The last generation of the HS (Harmonic Focus) is even more appropriate since it is approved for closing vessels up to 5 mm in diameter [30]. While a study by Leonard and Timon [31] concluded that use of a HS was not superior to conventional techniques with respect to operative time, many other studies reported decreases in operative time of between 6 and 78 minutes [9, 10, 16, 23, 30–39]. Even in the thyroidectomy with central or lateral neck dissection, the use of HS significantly reduces the operating time [40, 41].

Several studies have demonstrated that the use of harmonic scalpels led to a decrease in postoperative drainage, which also prevents postoperative surgical site infections. Similarly, we found the amount of postoperative drainage in patients treated with harmonic scalpels was reduced with respect to the other group. The reduction in intraoperative bleeding allows a more precise control of small vessels, which contributes to of the reduction in postoperative drainage. Surgeons preferred not to place drains in cases with low intraoperative bleeding; the number of these cases was higher in the HS group [32, 35, 42, 43].

The major complications of thyroid surgery are RLN palsy and hypocalcemia. An important issue concerning the use of new sealing modalities is the extent of lateral thermal conduction and associated tissue injury. Some authors have attributed these two complications to the lateral thermal effect of harmonic excitation [16, 23, 33]. Several studies in the literature, however, show that harmonic scalpels can be used safely in thyroid surgery with no increase in the number of complications [44–46]. In four trials, transient symptoms of hypoparathyroidism occurred more frequently in the conventional group that in the HS group [26, 32, 35, 42]. Our results seem to support the hypothesis that the reduced tissue injury resulting from less heat generated by the HS might lead to a reduced risk of impaired vascularity in the parathyroids glands. Randomized studies in future prospective with a larger number of patients are needed to draw more meaningful conclusions paying attention to the influence of HS use on complications after thyroid surgery.

In the literature, the postoperative pain has been rarely examined. The pain intensity at the surgical site was reported in three trials. While a study of Cordón et al. showed no statistical significance between the HS and conventional group [42], the trials of Defechereux et al. and Miccoli et al. demonstrated significant differences between the two groups regarding the VAS and VRS pain assessment and the mean consumption of analgesics [32, 35]. A possible explanation is that the HS causes reduced tissue injury, with no neuromuscular stimulation, as would be induced by electrocautery.

A major criticism to HS comes from its cost: it is disposable and expensive. The contract price for the disposable items and generator vary across different health authorities [23, 47]. Anyway, according to our experience, the absence of metal clips sutures or ties and a quicker turnaround time permit on average to operate one more patient per list; moreover, the costs related to the correction of hypocalcaemia are remarkably reduced. That allows considering HS a cost effective device. More detailed studies have to be planned in order to precisely analyze the cost-effectiveness of this instrument.

## 5. Conclusions

In total thyroidectomy, HS is a reliable and safe tool. Its use is more effective than CH technique. The surgical operative time is shorter and the total drainage fluid volume is reduced; furthermore, the postoperative pain is less and the rate of transient hypocalcemia is lower. Both techniques are equivalent concerning RLN injuries and hospital stay.

## Figures and Tables

**Table 1:** Demographic characteristics and preoperative diagnosis in HS (Harmonic Scalpel) and CH (Conventional Hemostasis) groups.

	HS group (*n* = 50)	CH group (*n* = 50)
Age (years) (range)	48.7 (21–73)	51.4 (23–72)
Sex (M/F)	22/28	19/31
Thyroid volume (mean ± SD) (range), mL	41.3 ± 12.9 (11–62)	37.8 ± 16.1 (9–61)

Preoperative diagnosis		
Simple multinodular goiter	29	31
Toxic multinodular goiter	11	10
Graves disease	4	5
Differentiated carcinoma	6	4

**Table 2:** Operative and postoperative data in HS (Harmonic Scalpel) and CH (Conventional Hemostasis) groups.

	HS group (*n* = 50)	CH group (*n* = 50)	*P *value
Operative time (mean ± SD) (range), min	44.9 ± 8.3	69.5 ± 10.7	*P* < .001
Postoperative drainage at 24 h (mean ± SD) (range), mL	37.4 ± 2.4	56.1 ± 4.2	*P* < .001
Hospital stay (mean ± SD), days	2.2 ± 0.9	3.7 ± 1.3	*P* > .05, NS
Thyroid weight (mean ± SD) (range), grams	44.8 ± 19.7	51.1 ± 15.8	*P* > .05, NS

Postoperative pain			
VAS at 24 h	3.89 ± 1.07	5.82 ± 1.43	*P* < .001
VAS at 48 h	1.99 ± 0.97	3.69 ± 1.36	*P* < .001
VRS at 24 h	1.81 ± 0.75	2.15 ± 0.84	*P* < .001
VRS at 48 h	1.06 ± 0.78	1.74 ± 0.62	*P* < .001

Postoperative complications			
Transient hypocalcemia	7	21	*P* < .01
Definitive hypoparathyroidism	0	0	NS
Transient recurrent laryngeal nerve injury	0	2	NS
Permanent recurrent laryngeal nerve palsy	0	0	NS

SD: standard deviation; NS: not significant; VAS: visual analogic scale; VRS: verbal response scale.
